# Synthetic Applications of Aziridinium Ions

**DOI:** 10.3390/molecules26061774

**Published:** 2021-03-22

**Authors:** Jala Ranjith, Hyun-Joon Ha

**Affiliations:** Department of Chemistry, Hankuk University of Foreign Studies, Yongin 17035, Korea; ranjithreddyiict@gmail.com

**Keywords:** aziridine, nonactivated aziridine, aziridinium ion, ring-opening, synthesis, nitrogen-containing molecules, regio- and stereoselective

## Abstract

Nonactivated aziridine with an electron-donating group at the ring nitrogen should be activated to an aziridinium ion prior to being converted to cyclic and acyclic nitrogen-containing molecules. This review describes ways to generate aziridinium ions and their utilization for synthetic purposes. Specifically, the intra- and intermolecular formation of aziridinium ions with proper electrophiles are classified, and their regio- and stereoselective transformations with nucleophiles are described on the basis of recent developments.

## 1. Introduction

**Figure 1:**
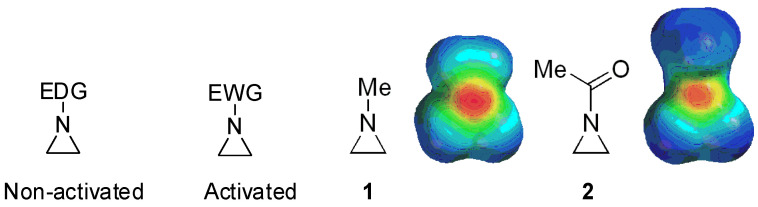
Chemical structures of “activated” and “nonactivated” aziridines and electrostatic potential maps of their representative examples *N*-methylaziridine and *N*-acetylaziridine. EDG = electron-donating group; EWG = electron-withdrawing group.

Aziridines are three-membered cyclic organic heterocyclic compounds with one nitrogen atom in the ring. They are valuable and versatile due to the reactive three-membered ring [1,2,3,4,5,6,7,8,9]. Highly ring-strained aziridines, e.g., other three-membered ring compounds such as cyclopropane and oxirane, render various nitrogen-containing compounds through ring-opening reactions with nucleophiles [10,11,12,13]. However, their stability and reactivity depend on substituents at the ring nitrogen (whether they are electron-withdrawing or -donating). Aziridines are bifurcated into “activated” ones bearing electron-withdrawing substituents at the ring nitrogen and “nonactivated” ones with electron-donating substituents [14] (Figure 1).

**Scheme 1:**
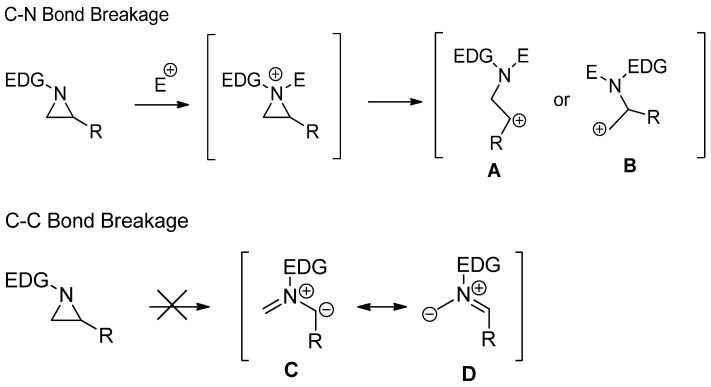
Comparison of two different ring openings via C–N and C–C bond cleavages of nonactivated aziridines.

Activated aziridines are quite reactive toward most nucleophiles, while nonactivated aziridines are inert unless they are activated as aziridinium ions or their equivalents by proper electrophiles including alkyl, acyl, trimethylsilyl, and Lewis acids [15,16]. When the simple aziridine without any substituent on the ring is protonated as an aziridinium ion, the ring strain is increased by 47 kJ/mol [17]. Most ring-opening reactions of nonactivated aziridines proceed with breakage of the bond between C and N, such as **A** or **B**, with the assistance of electrophiles after the formation of aziridinium ions. Experimental and theoretical studies have shown that nonactivated aziridine is possibly not serving as a 1,3-dipole, as shown in **C** and **D**, while activated aziridines can be utilized for a cyclization reaction with cleavage of the C–C bond for dipolarophile [17,18,19]. This difference is due to bond energy differences between C–N and C–C of nonactivated aziridines (Scheme 1).

**Figure 2:**
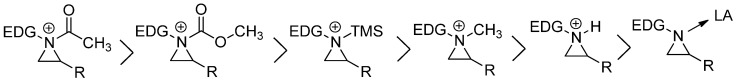
Order of reactivities of aziridinium ions by different electrophiles.

Thus, most reactions with nonactivated aziridines are carried out with the formation of aziridinium ion as a quaternary ammonium at first, which is created via the chemical bond between nucleophilic and basic ring nitrogen and an applied electrophile [15,16]. The aziridinium ion as an ionic intermediate is a well-known chemical species called “nitrogen mustard”, including the notorious chemical warfare agent VX [*O*-ethyl *S*-[2-(diisopropylamino) ethyl] methylphosphonothioate [20]. In most cases, this aziridinium ion intermediate has a classical ammonium ion character, albeit strained [21]. However, there are many ways to generate aziridinium ions with various electrophiles. Extensive calculation showed that reactivity differences were ordered as shown in Figure 2. Acyl aziridinium is the most active one, followed by alkoxycarbonyl, trimethylsilyl, alkyl, and protonyl aziridinium ions. The lewis acid-coordinated complex is the least active one [14].

**Scheme 2:**
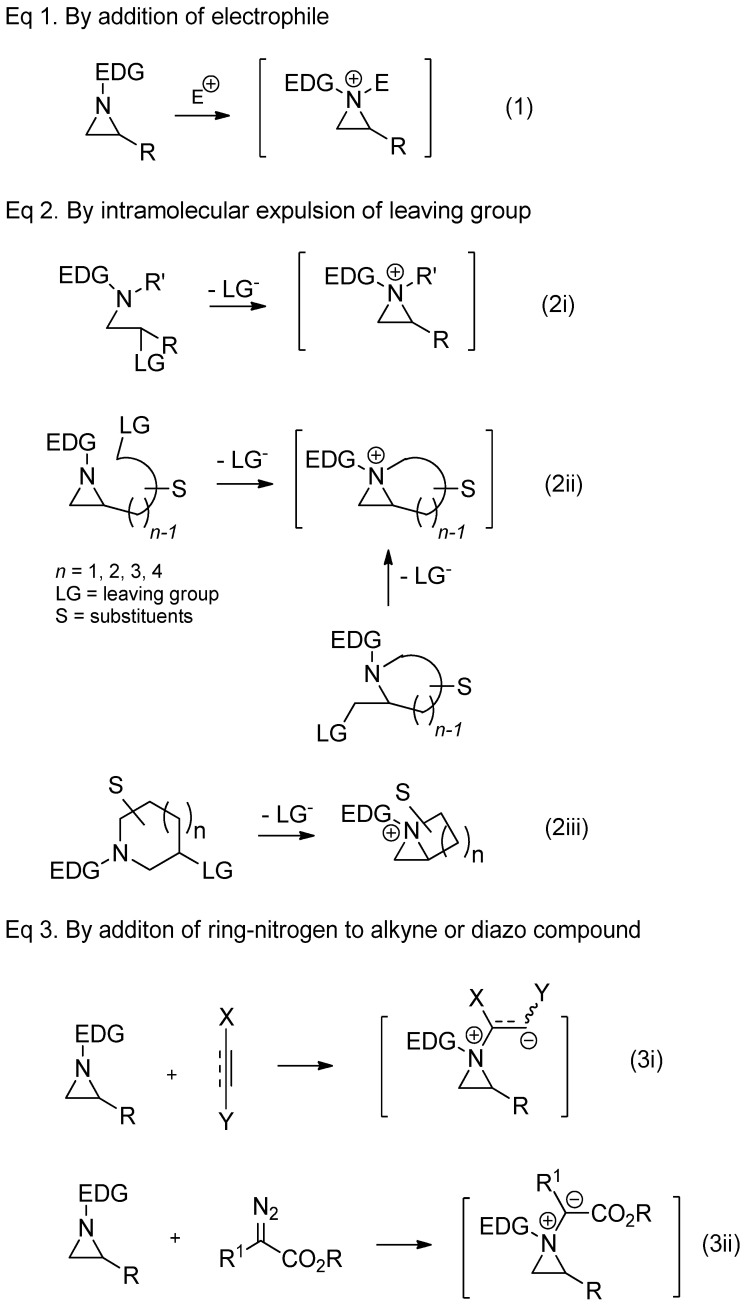
General scheme for the formation of aziridinium ions as synthetic intermediates from several different types of starting substrates.

There are several protocols to generate aziridinium ions from nonactivated aziridines, as shown in Figure 2. Direct addition of external electrophiles is the most convenient and easiest way for the preparation of an aziridinium ion as a quaternary ammonium salt. Electrophiles needed for correspondents shown in Figure 2 include acid halides, haloformates, trimethylsilyl halide, halomethane, proton, and Lewis acid (Equation (1), in Scheme 2). Aziridinium ions can also be generated upon expulsion of the leaving group at the β-position of amine of the acyclic starting substrate (Equation (2i), in Scheme 2) [18,19]. The same strategy to expulse the leaving group from the pendant of aziridine or 2-leaving group substituted methyl aza-cycle yielded a bicyclic aziridinium ion (Equation (2ii), in Scheme 2) [22,23]. The same bicyclic aziridinium ion can also be derived from removal of the leaving group at the β-site of cyclic starting substrates through aza-ring contraction (Equation (2iii), in Scheme 2) [24,25]. Lastly, aziridinium ylides schematized below (Equations (3i) and (3ii), in Scheme 2) can be created by addition of a ring-nitrogen to alkyne (Equation (3i), in Scheme 2) or diazo compound (Equation (3ii), in Scheme 2).

**Scheme 3:**
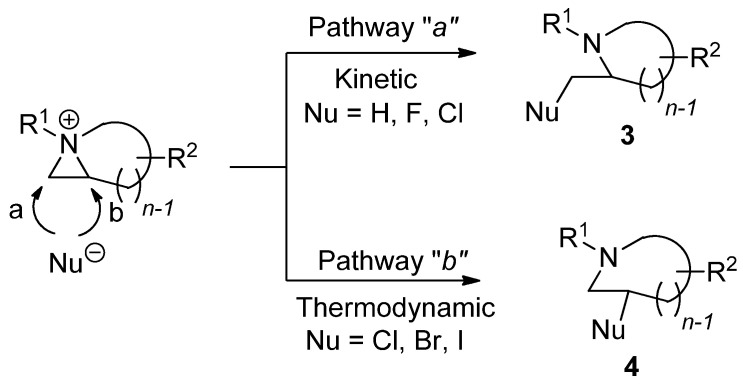
Two different ring-opening reaction pathways of nonactivated aziridine: “*a*” (kinetic) and “*b*” (thermodynamic) with the formation of an aziridinium ion with electrophile.

Chemical properties including stability and reactivity are dependent on characteristics of aziridinium ions. The way to make an aziridinium ion using an electrophile is the key to lead to the reaction of a nonactivated aziridine. The formation of aziridinium ions is sometimes observed spectroscopically. However, experimental and theoretical evidence has taught us that the following ring-opening reaction from an aziridinium ion is known to have a single transition state without forming any intervening ground state [26]. Different starting substrates and applied electrophiles can diversify the reaction products depending on whether the reaction proceeds via pathway “*a*” or “*b*”. The regiochemical pathway of ring opening via “*a*” is kinetic while the ring opening via “*b*” is thermodynamic, yielding reaction products **3** and **4** as regioisomers, respectively (Scheme 3).

The two different regiochemical pathways are bifurcated into kinetic and thermodynamic pathways with substituents attached to the aziridine ring [27,28]. When 2-substitued nonactivated aziridine is activated and then treated with various nucleophiles in the same medium, some nucleophiles such as bromine and iodine can lead the reaction into the thermodynamic pathway, while hydride and fluorine yield the kinetic product. In particular, when the hydride nucleophile is applied, only a kinetic product is afforded without formation of the regioisomer. The nucleophile “chlorine” behaves in between, showing that the kinetic product is formed first. It is then diminished with elapsed time to give the thermodynamic product from the equilibrated aziridinium ion intermediate [29]. This big regiochemical difference can also be observed between acetate and water, although both are oxygen nucleophiles that can attack aziridinium ions [22,23]. The reactivity is also influenced by the solvent as in the case of most nucleophilic substitution reactions. The medium effects are also quite big for ring-opening reactions of nonactivated aziridines due to different activation energies [15,16].

## 2. Synthetic Application of Aziridinium Ions

Many studies have reported synthetic applications of aziridines to provide a versatile entry to various nitrogen-containing molecules. In addition, recent advances in preparative enantiopure nonactivated aziridines warrant a streamlined synthesis of biologically important molecules, including natural products such as aza-sugars, alkaloids, and others [30,31,32]. However, a few problems need to be solved. Firstly, an efficient and general method is needed to prepare starting aziridines bearing diverse substitutes at three different sites, including N1 nitogen and two carbons, C2 and/or C3 [33,34,35,36]. For a more efficient and diverse use of nonactivated aziridines, a better understanding of the regiochemical pathway is needed. It is known that the regiochemical pathway of nonactivated aziridine is more diverse than that of activated aziridine [1,2,3,4,5,6,7,8,9]. In this short review, we describe synthetic developments to build important nitrogen-containing cyclic and acyclic molecules according to a few reports published recently.

### 2.1. Aziridinium Ions by Addition of External Electrophiles

**Scheme 4:**
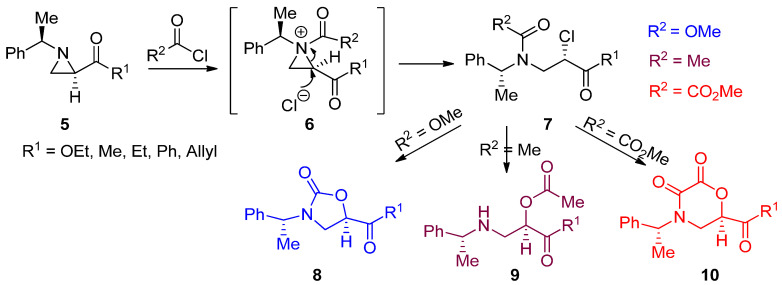
Reactions of 2-acylaziridines with acid chlorides, specifically including methyl chloroformate, acetyl chloride, and methyl chlorosuccinate.

The most popular and general synthetic method to generate an aziridinium ion is by adding electrophiles to nonactivated aziridines, as shown in Eq 1 of Scheme 2. Representative examples are described below. When chiral (2*R*,1′*R*)-2-acyl-(1′-phenylethyl)aziridines (**5**) and chiral nonactivated aziridines were treated with various acid chlorides such as acetyl chloride, methoxymethyl formate, and oxalyl chloride, the corresponding *N*-acylaziridinium ion (**6**) intermediates were generated by acylation of the nucleophilic aziridine ring nitrogen. These *N*-acylaziridinium ion intermediates then reacted with chloride released from the acid chloride, an electrophile, to give rise to ring-opened β-amino-β-chlorocarbonyl compounds (**7**). When we carried out the reaction in CHCl_3_, chlorinated compounds were observed [37]. However, under most reaction media including CH_3_CN, subsequent displacement of chloride with an internal oxygen nucleophile from methylchloroformate, acetyl chloride, and methyl chlorooxoacetate proceeded, yielding oxazolidin-2-ones (**8**), β-amino-α-acetyloxypropionates (**9**), and morpholin-2,3-diones (**10**), respectively (Scheme 4) [38].

**Scheme 5:**
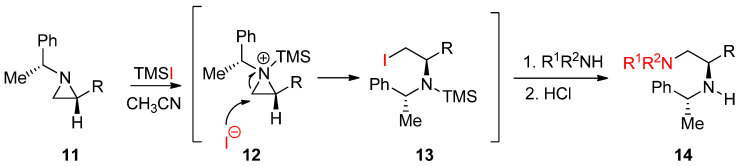
Formation of the trimethylsilyl (TMS) aziridinium ion with nonactivated aziridine and its subsequent ring opening by iodine and amine for the synthesis of 1,2-diamines.

Another useful external electrophile applicable to nonactivated aziridines is trimethylsilyl iodide with the formation of an aziridinium ion attached to the ring nitrogen as shown in Scheme 5. When enantiopure (1′-phenylethyl)aziridines (**11**) were treated with TMSI, the aziridinium ion (**12**) was formed. Subsequent ring opening by the released iodide gave rise to an iodinated product (**13**), whose iodine was replaced by amine to afford enantiopure diamines (**14**) (Scheme 5) [39,40].

This method is quite good to introduce a mild nucleophile that is not reactive enough to break the aziridine ring. In addition, TMS used for the activation of aziridine through an aziridinium ion is removed after addition of a nucleophile to replace iodine when the reaction proceeds. This method has been used for the synthesis of many biologically important molecules in our lab [40]. 

**Scheme 6:**
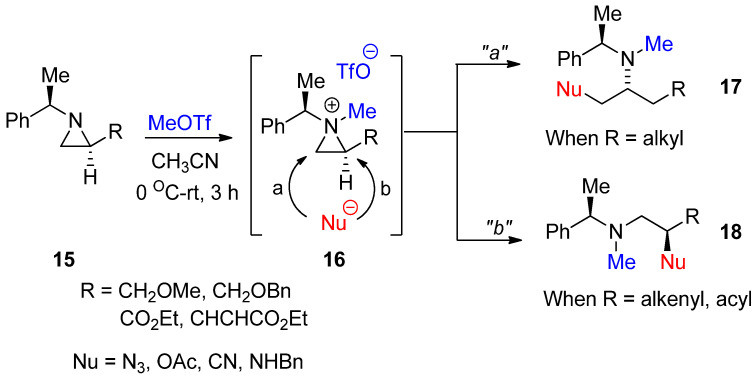
General scheme for “*N*-methylative aziridinium ring opening” of nonactivated aziridines.

As shown in Scheme 1, an aziridinium ion for the activation of nonactivated aziridine is generated by adding an alkyl group to aziridine nitrogen with the formation of quaternary amines. However, there is not an efficient method to introduce an alkyl group without breakage of the highly ring-strained aziridine ring. In addition, this aziridinium ion as a quaternary amine formed by an external alkyl group should be inert so that it does not react with the counter anion of electrophiles after alkylation of the amine, i.e., the counter anion of the ammonium ion should not have the nucleophilicity to break down three-membered ammonium ions. We successfully achieved formation of a methylated aziridinium ion (**16**) by treating starting aziridine (**15**) with methyl triflate, taking advantage of the extremely high nucleofugality of the triflate anion in a highly efficient manner. The formation of methylated aziridinium ion was observed using ^1^H- and ^13^C-NMR spectra. This methylated aziridine easily reacted with proper nucleophiles, whose regiochemical pathways were dependent on starting substrates. With simple alkyl substituents R, the ring opening proceeded via pathway “*a*” for **17**, while the starting material with vinyl or acyl at R gave product (**18**) from ring opening via pathway “*b*” (Scheme 6) [41]. This was the first study to generate aziridinium ions by methylation. We call this reaction “*N*-Methylative aziridinium ring opening”. This methylated aziridinium ion using MeOTf is stable. Such ring openings are possible with many external nucleophiles such as acetoxy, azide, hydride, hydroxy, and nitrile to realize the *N*-methylamino alkyl products. They provide easy excess to target molecules. A big advantage of this method is that *N*-methylamino compounds can be generated without an extra reaction to introduce a methyl group at the nitrogen of target molecules, if necessary.

**Figure 3:**
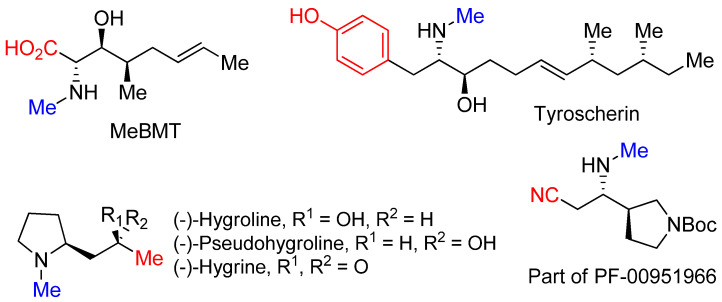
Molecules synthesized from “*N*-methylative aziridinium ring opening” of nonactivated aziridines.

This “*N*-methylative aziridinium ring opening” gives us an opportunity to obtain biologically important molecules including MeBMT [42], tyroscherin [43], hygrolines, and their analogous alkaloids [44], as well as a structurally important part of drug candidate PF-00951966 [45], as shown in Figure 3.

**Scheme 7:**
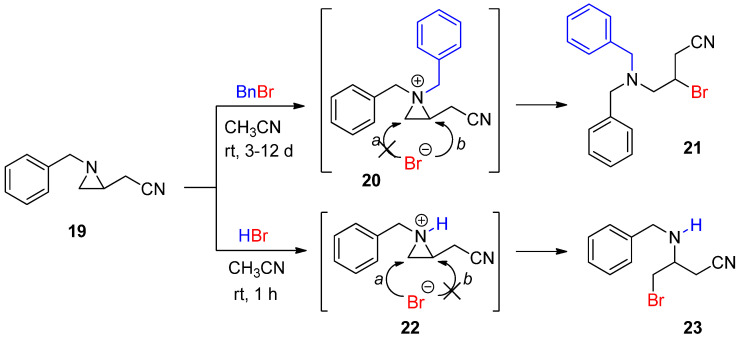
Ring-opening reactions of *N*-benzylaziridine as a nonactivated aziridine by BnBr and HBr with formation of the aziridinium ions (**20** and **22**) and their products (**21** and **23**).

This method of “*N*-methylative aziridinium ring opening” can be expanded to other alkyl groups to be “alkylative aziridinium ring opening”, including ethyl, allyl, and so on. Such studies are in progress in our lab. Benzylation of aziridine to yield a benzylated aziridinium ion was successfully achieved by treatment with benzyl bromide, whose bromine was released and reacted to give a brominated product. Its regiochemical outcome was derived from the thermodynamic pathway. When the same substrate was treated with HBr, a protonated aziridinium ion was generated, and subsequent ring opening occurred at C3 without any substituent, assuming that the regiochemical pathway was dominated by the kinetic coordinate. These two contrasting regiochemical pathways stemmed from different aziridinium ions by the addition of a benzyl group and a proton as electrophiles to the same aziridine (Scheme 7) [46]. This distinctive regiochemistry taught us that it would be possible to control reaction pathways according to the formation mechanism of the aziridinium ion [47,48].

Many studies have reported the regioselectivity of ring-opening reactions of nonactivated aziridinium ions via pathway “*a*” (nonsubstituted carbon C3, kinetically favored) or “*b*” (substituted carbon C2, thermodynamically favored) depending on chemical aspects. When the aziridine ring has an allylic or benzylic C2–N1 bond as in 2-vinyl or 2-phenylaziridines, ring-opening reactions proceed at the C2 position (pathway “*b*”) regardless of electrophiles or nucleophiles. Ring opening with 2-acylaziridines takes pathway “*b*” with a few exceptions of electrophiles and nucleophiles applied [47,48]. With 2-alkylaziridines, their reaction pathways are diversified depending on the nature of electrophiles and nucleophiles. However, the reaction would warrant a kinetically controlled ring-opened product at C3 (pathway “*a*”). According to experimental and theoretical data, including ours, a general overview is provided in Table 1 as a practical guide to predict the regiochemical pathway of nucleophilic nonactivated aziridines using different substrates, electrophiles, and nucleophiles. Bearing this in mind, it might also be possible to predict a regioselective preference for unexamined ring-opening reactions of nonactivated 2-substituted aziridines [49].

**Scheme 8:**
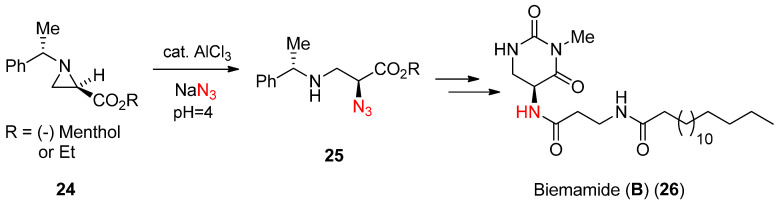
Azide-induced ring opening of enantiopure *N*-phenylethylaziridine-2-carboxylate and its use for the synthesis of biemamide (**B**).

In the literature, there are ample examples for the activation for ring-opening reactions using a Lewis acid, one of which is shown in Scheme 8. This scheme showed that an azide nucleophile could drive the aziridine ring (**24**) with assistance of Lewis acid AlCl_3_ in aqueous medium to yield α-azido-β-aminopropionate (**25**) [50], which was utilized for the synthesis of natural product biemamide (**B**) (**26**) [51].

### 2.2. Aziridinium Ions upon Expulsion of the Leaving Group

**Scheme 9:**
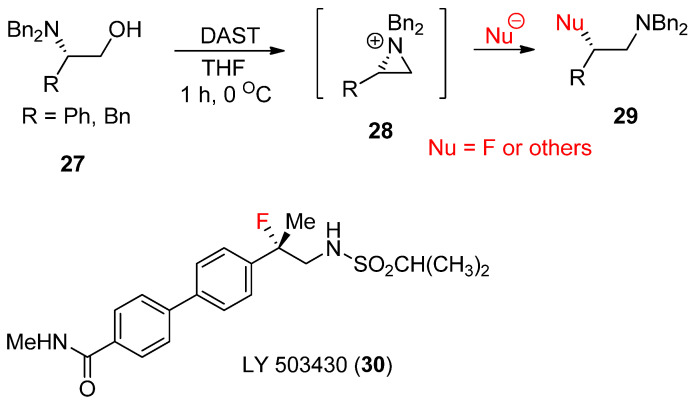
Formation of aziridinium ion from β-hydroxyamine followed by nucleophilic ring opening in the presence of cat trifluoroacetate.

For the last few years, Cossy’s group has extensively studied the synthesis of various compounds from the formation of aziridinium ions, some of which were reported in a review article [52,53]. A typical method for aziridinium ions comes from expulsion of the leaving group of hydroxides at β-hydroxy amines [54]. One big advantage of β-hydroxyamine is that its enantiopure form is readily available from a rich chiral pool of amino acids. Its stereoselective transformation can realize diverse enantiopure products starting from diverse chiral amino acids [55]. Transforming the alcohol moiety into a good leaving group has allowed the rearrangement of these β-amino alcohols (**27**) to yield the aziridinium ion (**28**), which can then readily react with a large number of nucleophiles to afford ring-opened products (**29**). An overview of recent progress realized for the rearrangement of these β-amino alcohols in the presence of catalytic amount (CF_3_CO)_2_O and H_2_SO_4_ or *N*,*N*-diethylaminosulfur trifluoride (DAST) has been reported [56]. This method has been applied for the synthesis of drug candidate LY-503430 (**30**) for Parkinson’s disease (Scheme 9) [57].

**Scheme 10:**
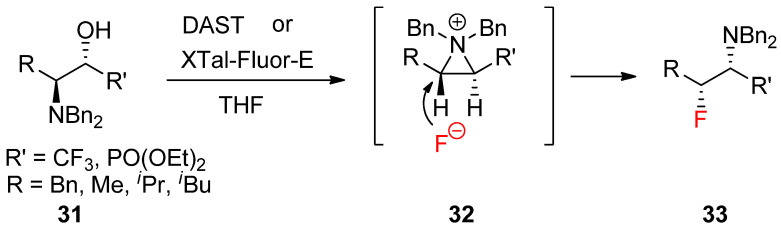
Preparation of α-trifluoromethyl amines or α-amino-β-fluorophosphonates bearing extra fluorine at the α-position from the kinetically favored fluoride attack of an aziridinium ion intermediate formed from β-amino-α-trifluoromethyl alcohols.

A similar reaction with the fluoride nucleophile yielded a fluorinated product after the formation of an aziridinium ion driven by DAST [58]. The synthesis of various optically active α-trifluoromethyl amines (**33**) was realized from β-amino-α-trifluoromethylalcohols (**31**) via an aziridinium ion intermediate (**32**) under a kinetic condition (Scheme 10) [59].

Recently, a report revealed that the same strategy is applicable to realize stereoselective and regioselective synthesis of α-amino-β-fluorophosphonates [60]. 

**Scheme 11:**
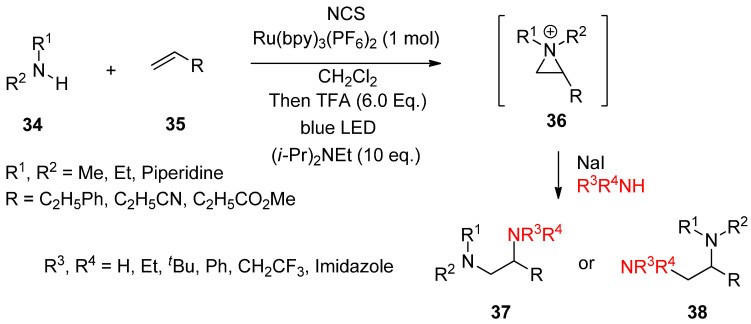
Photo-induced single-electron transfer for olefin-diamination with alkylamines.

Most aforementioned cases for the formation of an aziridinium ion took ionic pathways. Recently, photo-induced single-electron transfer for olefin-diamination with alkylamines was successful (Scheme 11) [61]. A useful protocol to introduce two different amines at each site of olefin with an aziridinium ion as an intermediate was introduced [61].

The synthetic protocol consists of the formation of an aziridinium ion (**36**) from an amine (**34**) and olefin (**35**) via in situ activation with *N*-chlorosuccinimide (NCS), protonation, and photo-induced SET (single-electron transfer) reduction in the presence of a Brønsted acid and Ru(bpy)_3_(PF_6_)_2_ (1 mol%) as the photocatalyst in CH_2_Cl_2_ solvent under blue light irradiation at 0 °C. The following ring opening by another amine gives rise to vicinal diamine compounds (**37**, **38**).

### 2.3. Bicyclic Aziridinium Ions from Substituted Aziridines

**Scheme 12:**
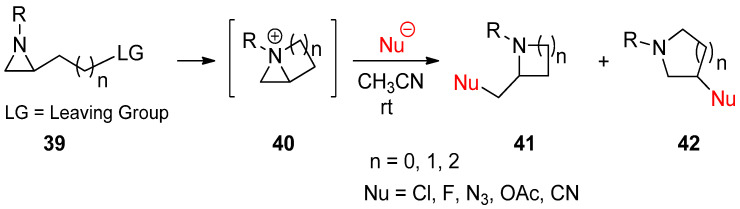
Generation of bicyclic aziridinium ions (**40**) from displacement of a suitable leaving group at the aziridine side chain in **39** and subsequent ring-opening reactions to yield regioisomers (**41** and **42**).

Instead of applying an external electrophile, aziridinium ions can be generated as bicyclic forms (**40**) through displacement of a suitable leaving group at the side chain, as shown in (**39**) by nucleophilic aziridine amine (Scheme 12) [62]. Ring openings of these bicyclic aziridinium ions may occur due to nucleophilic attacks either at the bridgehead or at bridge positions of aziridine to afford substituted aza-rings with substituents without loss of the substrate’s stereochemistry. The regiochemistry was determined on the basis of the characteristics of the nucleophile [63,64]. More specifically, bromide and iodide attached to the bridgehead, while nitrile and acetate favored the bridge position [22,23,64].

**Figure 4:**
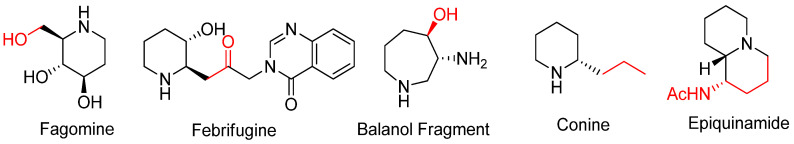
Representative examples synthesized from Scheme 12 as a key step.

This convenient approach renders the construction of various types of aza-rings with substituents from nucleophilic attacks either at the bridgehead or at bridge positions of bicyclic aziridinium ions without loss of the substrate’s stereochemistry [65]. This synthetic strategy can be used to obtain many biologically active alkaloids and their analogues including fagomine, febrifugine, balanol, conine, and epiquinamide, as shown in Figure 4 [22,23,31,66].

### 2.4. Bicyclic Aziridinium from Ring Contraction of Azaheterocycles

**Scheme 13:**
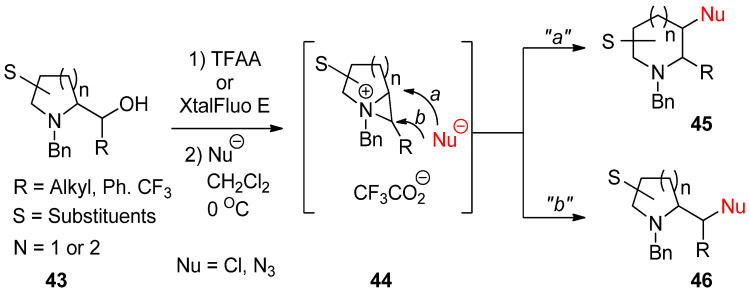
Formation of bicyclic aziridinium ion from aza-ring by removing the hydroxy group of hydroxymethyl at the α-position and the subsequent ring opening by a nucleophile either at the suitably disposed bridgehead (***a***) or at the bridge position (***b***) to lead products.

An aza-ring (**43**) with an appendage of hydroxymethyl at the α-position of amine engenders the bicyclic aziridinium ion (**44**) with removal of the leaving group including a hydroxy group. This was further reacted with an applicable nucleophile to yield new aza-ring products (**45** and/or **46**) via ring opening at the bridgehead (*a*) or the bridged (*b*) position of the bicyclic aziridinium ion (**44**) (Scheme 13). Regarding the regiochemical pathway, whether the reaction proceeds via pathway “*a*” or “*b*” depends on nucleophiles [67,68,69].

**Figure 5:**
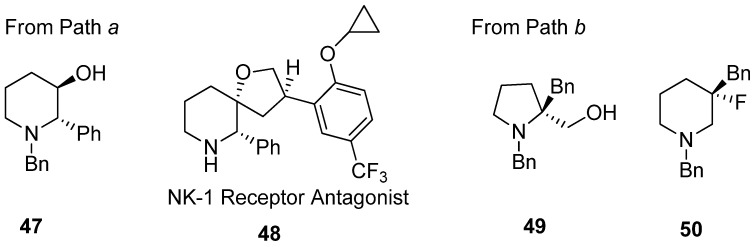
Representative examples of Scheme 13 following pathway “*a*” or “*b*” to prepare various aza-cyclic valuables.

Application of various nucleophiles through pathway “*a*” or “*b*” gives an efficient synthetic strategy to prepare various aza-cyclic valuables (Figure 5). Following pathway “*a*”, nonpeptidic NK-1 receptor antagonist L-733060 (**48**) was realized starting from 3-hydroxy-2-phenyl piperidine (**47**) [56,70]. Most cases with diethylaminosulfur trifluoride (DAST) have yielded enlarged fluorinated piperidine ring compounds (**50**) from hydroxymethylpyrrolidine (**49**) via fluorine-driven ring opening in a stereoselective manner [71,72].

**Scheme 14:**
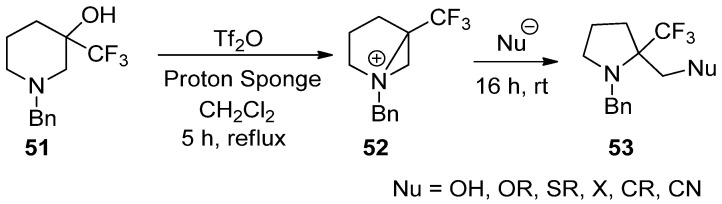
Preparation of 2-substituted methyl 2-(trifluoromethyl)pyrrolidines from ring contraction via bisaziridinium ion with removal of the hydroxy group of 3-hydroxy-3-trifluoromethylpiperidine.

This reaction, generation, and ring opening of a bisaziridinium ion (**52**) from 3-hydroxy-3-trifluoromethyl piperidine (**51**) with removal of the hydroxy group could also be applicable for the synthesis of biologically important α-trifluoromethyl pyrrolidines (**53**) (Scheme 14) [73,74].

### 2.5. Aziridinium Ylides

**Scheme 15:**
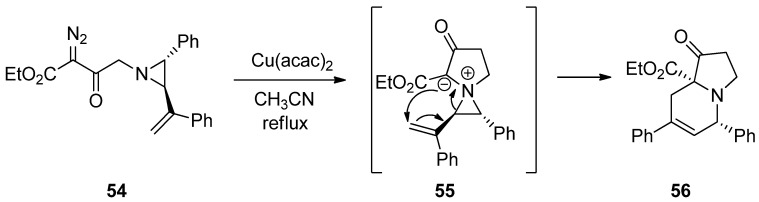
Formation and ring expansion of aziridinium ion generated from intramolecular Cu(II)-catalyzed aziridinium ylides.

**Scheme 16:**
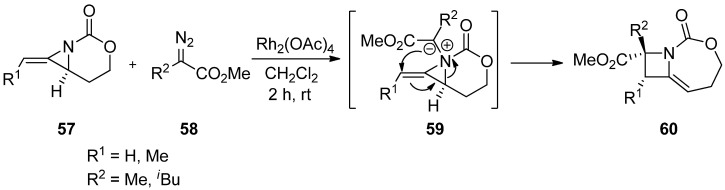
Formation and ring expansion of aziridinium ion generated from intermolecular Rh-catalyzed aziridinium ylides.

Recently, aziridinium ylides were developed by the addition of electrons at the aziridine nitrogen in the starting substrates to carbene or alkyne, followed by subsequent addition of an anion located at the α-position of the carboxylate to the olefin to afford new rings via intramolecular ring-opening pathways. The schemes below (Scheme 15 and Scheme 16) show the formation of aziridinium ylides (**55** and **59**) via intramolecular or intermolecular addition of diazoacetate (**54** or **58**) to aziridine (**54** or **57**) with metal catalysts. Subsequent ring openings initiated by the anion at the α-position of carboxylate give ring-expanded products (**56**) and (**60**) [75,76,77].

**Scheme 17:**
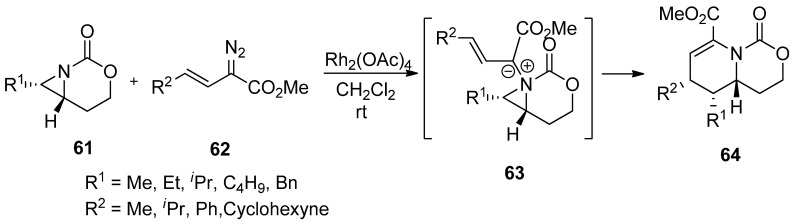
Ring expansion of aziridines via aziridinium ylides to dehydropiperidines.

The addition of carbene generated by Rh in an intermolecular manner was reported by Rowland with limited examples [78]. This has been developed to a great extent by Schomaker’s group, whose representative example is shown in Scheme 17 [79]. The formation of aziridinium ylide (**63**) was created by adding carbene from diazoacetate (**62**) to aziridine (**61**)**,** whose aziridine ring was opened to give an expanded aza-ring (**64**).

**Scheme 18:**
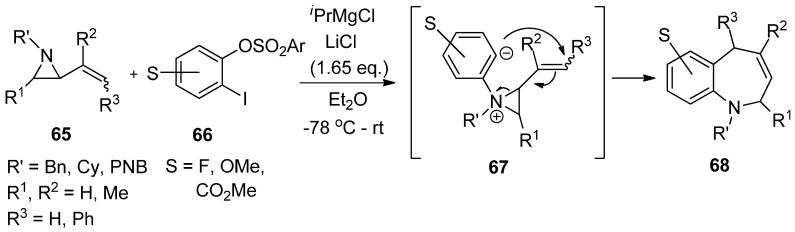
Formation of aziridinium ion by adding aryne to aziridine and subsequent strain-release cycloaddition reaction.

Another interesting formation of aziridinium ylide (**67**) was reported by Yudin’s lab using the reaction of aryne from 1-iodo-2-sulfonyloxybenzene (**66**) with 2-alkenylaziridine (**65**). This was followed by anionic rearrangement to produce benzazepine (**68**) with a good yield (Scheme 18) [80].

**Scheme 19:**
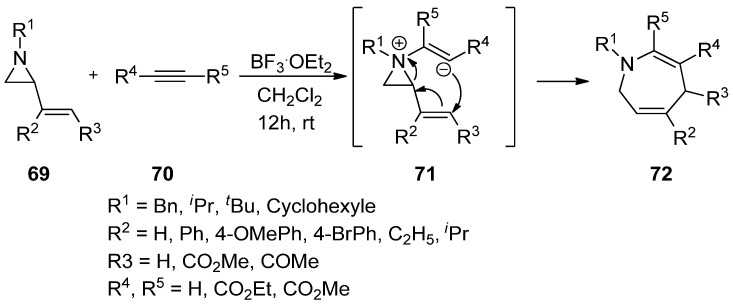
Addition of aziridine-nitrogen to alkyne to form aziridinium ion and its release to give azopanes.

It has been disclosed that the addition of electron-rich nitrogen at the nonactivated aziridine (**69**) to alkyne (**70**) can make an aziridinium ylide (**71**) whose anionic olefin can attack the vinyl group, thus giving rise to various substituted benzazepines (**72**) (Scheme 19) [81].

**Scheme 20:**
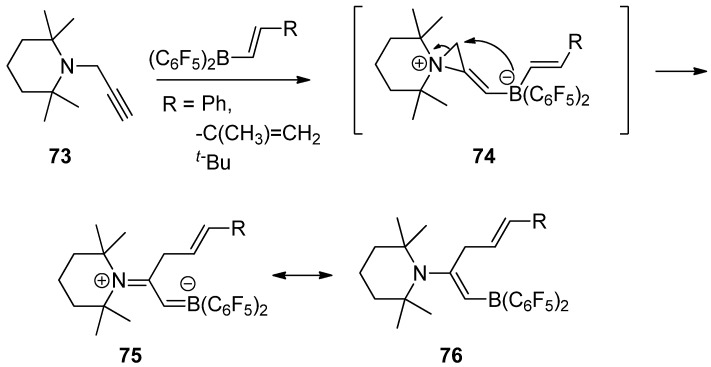
A reactive zwitterionic aziridinium intermediate from the reaction of *N*-propargyltetramethylpiperidine with *trans*-alkenyl-B(C_6_F_5_)_2_ compounds and subsequent alkenylborate attack with ring opening to the activated three-membered aziridinium ion.

Recently, an interesting reaction was developed to generate a reactive zwitterionic aziridinium intermediate (**74**) from the reaction of *N*-propargyltetramethylpiperidine (**73**) with *trans*-alkenyl-B(C_6_F_5_)_2_ compounds via *trans*-1,2-amine/borane addition to a carbon–carbon triple bond. Subsequent alkenylborate attack with ring opening to the activated three-membered aziridinium ion afforded a stable boronated alkenyl piperidine resonance between (**75**) and (**76**), as shown in Scheme 20 [82].

## 3. Conclusions

Nonactivated aziridines are valuable starting materials for the synthesis of densely functionalized nitrogen-containing compounds in a highly regio- and stereocontrolled manner. Their chemical diversity stems from the intrinsic properties of nonactivated aziridines to be activated with electrophiles including acyl halide, TMS halide, alkylhalide, sulfonyloxyalkanes, protic acid, and a Lewis acid. In addition to the backbone of starting substrates and applicable nucleophiles, the chemical reactivity and regiochemical pathways depend highly on the electronic characteristics of aziridinium ions. In this review, we summarized the ways of generating and classifying various types of aziridinium ions for synthetic purposes. The hope is that readers find the value of nonactivated aziridines and their synthetic utilities.

## Figures and Tables

**Table 1:** Regioselectivity in ring opening of nonactivated 2-substituted aziridines either at C2 (pathway “*b*”) with a substituent or at C3 (pathway “*a*”) without any substituent depending on the substrate, the electrophile, and the nucleophile.

	E^+^	Lewis Acid	RCO^+^	H^+^	R^+^	TMS^+^
R^1^						
–CH=CH_2_, –CH=CH–COOEt		C2^c^	C2	C2 ^c^	C2	C2
Aryl		C2	C2	C2	C2 ^c^	C2 ^c^
COR, COOR, CONH_2_		C3	C2	C2	C2	C2
Alkyl		C3	C3 and/or C2 ^a^	C3	C3 ^b^	C3

^a^ Depending on the nucleophile (e.g., azide attacks at C2; alcohol attacks at C3). ^b^ Among halides, only the fluoride ion attacks the C3 position to a major extent. ^c^ Proposed regioselectivity (no experimental data available).
